# The Absence of Gasdermin D Reduces Nuclear Autophagy in a Cecal Ligation and Puncture-Induced Sepsis-Associated Encephalopathy Mouse Model

**DOI:** 10.3390/brainsci13030478

**Published:** 2023-03-11

**Authors:** Wei Su, Zhenxing Xie, Xiangjun Bai, Zhanfei Li, Xinghua Liu

**Affiliations:** 1 Trauma Center/Department of Emergency and Trauma Surgery, Tongji Hospital, Tongji Medical College, Huazhong University of Science and Technology, Wuhan 430030, China; 2 Department of Critical Care Medicine, Tongji Hospital, Tongji Medical College, Huazhong University of Science and Technology, Wuhan 430030, China; 3 Department of Neurology, Tongji Hospital, Tongji Medical College, Huazhong University of Science and Technology, Wuhan 430030, China

**Keywords:** sepsis-associated encephalopathy, pyroptosis, GSDMD, nuclear autophagy, LaminB, LC3, p62, novel object recognition test, open field test, transmission electron microscopy

## Abstract

Sepsis-associated encephalopathy (SAE) is a common complication of sepsis, which is a life-threatening condition resulting from a dysregulated host response to infection. Pyroptosis, a pro-inflammatory mode of lytic cell death mediated by GSDMD (Gasdermin D), is involved in the pathogenesis of SAE. While autophagy has been extensively studied in SAE, the role of nuclear autophagy is not yet well understood. In this study, we aimed to investigate the involvement of pyroptosis and neural nuclear autophagy in the pathogenesis of SAE. We analyzed a CLP (cecal ligation and puncture)-induced SAE model in wild-type and GSDMD^−/−^ mice to gain insights into the underlying mechanisms. Here, we show that in sepsis, neural nuclear autophagy is extremely activated, and nuclear LaminB decreases and is accompanied by an increase in the ratio of LC3BII/I. These effects can be reversed in GSDMD^−/−^ mice. The behavioral outcomes of septic wild-type mice are impaired by the evidence from the novel object recognition test (NORT) and open field test (OFT), but are improved in septic GSDMD^−/−^ mice. In conclusion, our study demonstrates the activation of neural nuclear autophagy in SAE. The absence of GSDMD inhibits nuclear autophagy and improves the behavioral outcomes of SAE.

## 1. Introduction

In recent times, there has been increasing focus on the pyroptosis mechanism in relation to sepsis-associated encephalopathy (SAE) [1,2,3,4,5]. SAE refers to a type of brain dysfunction that occurs as a result of sepsis. The symptoms include confusion, disorientation, delirium, decreased level of consciousness, and memory problems [6,7]. Early recognition and treatment of sepsis and its associated encephalopathy is critical for reducing the risk of long-term neurological complications and improving the outcomes [8]. Pyroptosis is a type of programmed cell death that is associated with inflammation and is triggered by the activation of certain signaling pathways in response to certain stimuli, including bacterial and viral infections, as well as cellular stress [9,10,11,12,13]. Gasdermin D (GSDMD) is known to regulate pyroptosis through its interaction with caspase-1, a critical enzyme involved in inflammasome activation. Specifically, caspase-1 cleaves GSDMD, forming N-terminal GSDMD (GSDMD-N), which pores on the cell membrane and leads to the subsequent release of pro-inflammatory cytokines, including interleukin-1β (IL-1β) [14]. Pyroptosis contributes to the pathogenesis of SAE by promoting inflammation and activating microglia in the brain, which have been demonstrated to result in neuronal dysfunction and potentially contribute to the development and progression of SAE [4,15].

Nuclear autophagy selectively degrades the nucleus of a cell through the cellular mechanism of autophagy, which is essential for maintaining cellular homeostasis by eliminating abnormal nuclei, preventing genetic mutations, and promoting cell survival [16,17]. In nuclear autophagy, the nucleus is targeted by autophagosomes, double-membraned vesicles that are involved in the degradation of cellular components. The autophagosomes engulf the nucleus and then fuse with lysosomes, which contain hydrolytic enzymes that break down the contents of the autophagosomes [18]. Autophagy shows involvement in various cellular processes, such as the DNA damage response, cellular senescence, and cellular metabolism regulation [19,20,21,22]. Additionally, it implicates in the onset of several diseases, including cancer [23] and neurodegenerative disorders [24,25,26].

Several markers exist that specifically identify nuclear autophagy. LaminB, a type of intermediate filament protein, participates in the formation and maintenance of the nuclear envelope, ensuring the nucleus’s integrity and organization [27]. During nuclear autophagy, degradation targets LaminB, which is considered important in initiating and regulating the process [14]. LC3 aids in the recruitment and anchoring of the autophagosomal membrane to the cytoplasmic membrane, as well as in the elongation and closure of the autophagosome [28,29,30]. Interfering with LC3s function can hinder this process [31]. The autophagy of the nucleus is also marked by the formation of autophagosome–lysosome structures, where the nucleus is engulfed by autophagosomes and is subsequently degraded after fusing with lysosomes [4]. In the process of autophagy, p62 binds to ubiquitinated cargo and delivers it to the autophagosome for degradation [32]. Thus, p62 acts as a selective autophagy receptor, targeting ubiquitinated aggregates, organelles, and other cytoplasmic components for autophagic degradation [33]. Additionally, p62 interacts with the ubiquitinated chromatin-associated protein HMGB1 and targets it for autophagic degradation, leading to a reduction in chromatin condensation [34].

In addition to nuclear autophagy, mitochondrial damage is also observed in SAE, which manifests as a shorter and smaller mitochondria with a disrupted membrane structure [4,35,36,37,38]. Mitochondrial damage in SAE can trigger the production of reactive oxygen species (ROS) and activation of inflammatory pathways, ultimately leading to neuronal cell death and brain dysfunction [39,40]. Impaired energy metabolism, resulting in a depletion of cellular ATP, which is critical for normal brain function, is another consequence of mitochondrial damage [36]. Furthermore, mitochondrial dysfunction can contribute to the activation of apoptotic pathways, which worsen brain damage in SAE [41]. This damage may be a compensatory response to increased energy demands, and may contribute to cognitive and emotional dysfunction. It is unclear whether GSDMD is also involved in the mitochondrial damage in SAE, and it may potentially affect the behavior of SAE. Recently, an increasing number of preclinical research studies have suggested that the disruption of mitochondrial functions and the potential loss of mitochondrial stress resilience may have an impact on animal behaviors, including cognitive impairment, mood disorders, anxiety, and psychosis. Therefore, it is highly probable that such mitochondrial impairment could also be connected to cognitive function [42,43]. Furthermore, positive emotions enable animals to regulate behaviors [44].

Nuclear autophagy is observed in SAE but is poorly understood. A previous study reported that the nuclear LaminB1 acts as a substrate for nuclear autophagy in primary human cells [14], triggering the study of nuclear autophagy in mammals. In the non-canonical release of IL-1β, GSDMD-N localized to LC3-positive autophagosomes in neutrophils, suggesting that GSDMD-N plays a role in autophagy [10]. However, the role of GSDMD in the nuclear autophagy of SAE is unclear. In this study, we examine whether the absence of GSDMD improves the outcomes of the CLP-induced SAE in GSDMD^−/−^ mice and analyze the autophagy in the nucleus and cytoplasm.

## 2. Materials and Methods

### 2.1. Reagents

Antibodies used in this study are listed in Supplementary Table S1.

### 2.2. Animals 

Male C57BL/6 mice (8 weeks, 20–25 g) were obtained from the Experimental Animal Center of Tongji Medical College at Huazhong University of Science and Technology. The GSDMD^−/−^ mice were purchased from GemPharmatech Co., Ltd. The mice were kept in a temperature-controlled environment and were given standard food and unlimited access to water. The experimental animal ethics committee of Tongji Hospital, affiliated with Huazhong University of Science and Technology (TJH-201901022), approved all animal experiments and ensured that they were conducted in accordance with the ARRIVE guidelines [45] and the National Institutes of Health guidelines for laboratory animal care.

### 2.3. CLP Model of Sepsis

Male C57BL/6 mice were subjected to cecal ligation and puncture to induce polymicrobial abdominal sepsis [46]. To anesthetize the mice, pentobarbital sodium was administered via intraperitoneal injection at a dosage of 50 mg/kg. Next, a 1 cm incision was made along the midline of the lower abdomen, followed by ligation of the distal cecum (approximately 1.5 cm) with a sterile 3.0 thread and puncturing using a sterile 21-gauge needle. Gentle expression of fecal matter was then carried out through the puncture site. Next, the intestines were placed back into the abdominal cavity and the incision was closed with sutures. In the sham-operated group, only the abdomen was opened and closed without any ligation or puncture being performed. After surgery, 300 uL of 37 °C sterile saline was injected subcutaneously to provide fluid resuscitation. The mice were randomly separated into four groups: the wild-type sham group; the wild-type CLP group; the GSDMD^−/−^ sham group; and the GSDMD^−/−^ CLP group.

### 2.4. Open Field Test (OFT)

The mice were subjected to a locomotor activity test three days after surgery. Each mouse was placed in the center of a square arena with dimensions of 40 cm in height, 50 cm in width and length, with a camera overhead. During the 5 min trial, the total distance traveled was recorded to evaluate locomotion. The arena was divided into 25 equal parts, and the amount of time spent in the border parts was determined as the border-zone duration. After each trial, the arena was thoroughly cleaned using 75% alcohol.

### 2.5. Novel Object Rrecognition Test (NORT)

One hour after the OFT, the mice were introduced into an arena containing two identical objects and were allowed to explore freely for 10 min. After a one-hour interval, the mice were returned to the arena where one of the objects had been replaced with a new one, and they were again given 10 min to explore. To eliminate any olfactory cues, the objects and the arena were cleaned thoroughly with 75% ethanol between each trial.

### 2.6. Immunofluorescence

The mice were anesthetized using pentobarbital sodium and perfused transcardially with 0.1 M phosphate-buffered solution (PBS), followed by 4% paraformaldehyde. Subsequently, the brain samples were fixed in a solution of 4% paraformaldehyde for 36 h, followed by a soak in 30% sucrose for 72 h. The brains were sliced into 30 μm-thick sections using a cryotome (Leica, Wetzlar, Germany) and then were treated with 0.5% Triton X-100, followed by blocking with 3% bovine serum albumin. Next, each brain slice was incubated with the relevant primary antibody overnight at 4 °C, and then incubated with a secondary antibody for 2 h at room temperature. The slices were finally stained with DAPI for 10 min. The images were captured using a fluorescence microscope (Mshot, Shenzhen, China). The antibodies used are listed in Supplementary Table S1.

### 2.7. Western Blotting

Cytoplasmic and nuclear fractions of brain tissues were extracted by using a Nuclear and Cytoplasmic Protein Extraction kit (Beyotime Biotechnology (Shanghai, China)). To obtain the whole-cell lysate, the brain tissue was homogenized with a RIPA lysis buffer that contained a protease inhibitor, then centrifuged at 12,000 RPM and 4 °C for 15 min. The protein concentration of each sample was determined using a BCA kit (Servicebio (Wuhan, China)). The proteins were separated using SDS-PAGE, then transferred onto PVDF membranes. After blocking with 5% bovine serum albumin, the membranes were incubated with specific primary antibodies overnight at 4 °C, followed by incubation with horseradish peroxidase-conjugated secondary antibodies for 1 h at room temperature. The protein bands were visualized using electrochemiluminescence (ECL) and the imaging system (Tanon 5200 (Shanghai, China)). The ImageJ software was used for optical density analysis.

### 2.8. Transmission Electron Microscopy (TEM)

The mice were anesthetized using pentobarbital sodium and perfused transcardially with 0.1M phosphate-buffered solution (PBS). Fresh prefrontal cortex tissues were harvested and immersed in 2% glutaraldehyde at 4 °C for 24 h. The tissues were then rinsed in 0.1 M PBS and fixed with 2% OsO4 at 4 °C for 60 min. After undergoing dehydration and embedding in resin, the tissues were cut into 700 nm-thick sections. The sections were stained with 4% uranyl acetate for 20 min and 5% lead citrate for 5 min. Images were scanned with a transmission electron microscope.

### 2.9. Measurements of Nuclear Distortions and Circularity

The immunofluorescence staining was performed on each group, as previously described [47]. The images were captured using a fluorescence microscope (Mshot, (Guangzhou, China)). The circularity of the nuclei was calculated using the ImageJ and by employing the formula: circularity = 4π (area/perimeter2).

### 2.10. Statistical Analysis

The data were expressed as the mean ± SEM. Statistical analysis was performed using GraphPad Prism 8. The results underwent the analysis of variance and the Newman–Keuls test, while the survival curve was analyzed through the Kaplan–Meier test and differences between groups were evaluated through a log-rank test. A *p*-value less than 0.05 was considered statistically significant.

## 3. Results

The absence of GSDMD improves the prognosis in mice with SAE.

**Figure 1:**
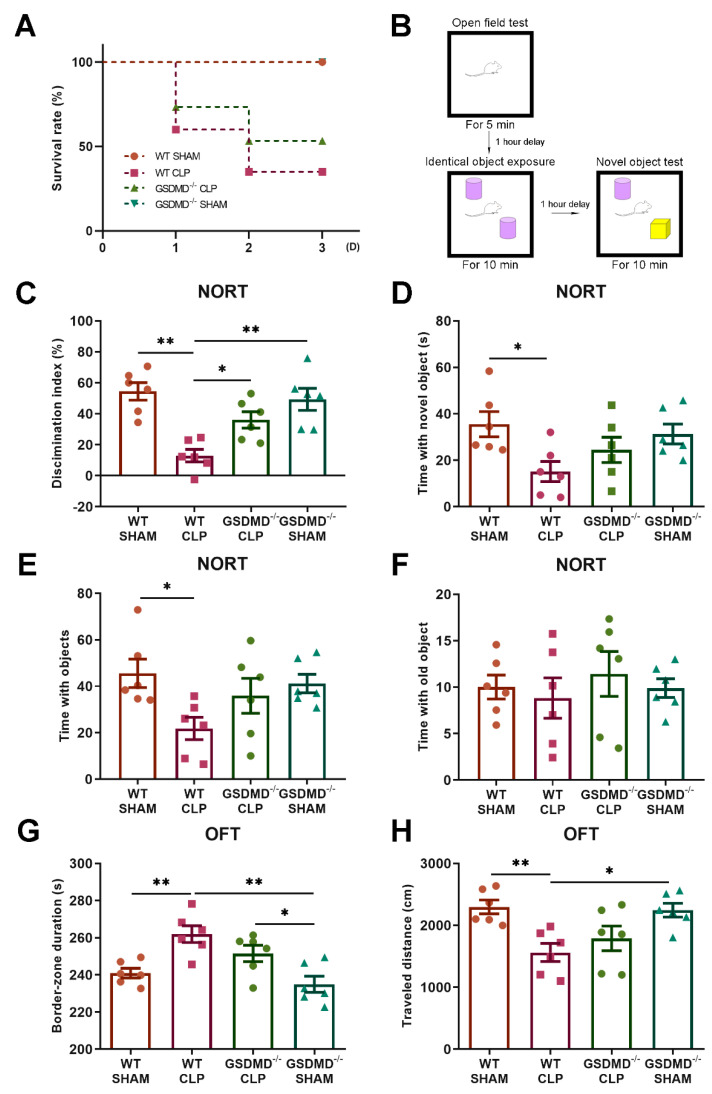
The absence of GSDMD improves the survival rate and behavioral outcomes of CLP-induced SAE in mice. (**A**) Survival curve. Seven of twenty wild-type CLP mice and eight of fifteen GSDMD^−/−^ CLP mice survived until day 3 after surgery. No mice died in the sham groups. (**B**) Schematic of OFT and NORT. (**C**−**F**) Discrimination index, time with novel object, time with objects, and time with old object in the NORT, respectively (n = 6 per group). (**G**,**H**) Border-zone duration and traveled distance in the OFT, respectively (n = 6 per group). * *p* < 0.05, ** *p* < 0.01.

The survival rates following CLP surgery in wild-type mice were 60% (12 out of 20) on the first day, 35% on the second day (7 out of 20 mice), and 35% on the third day (7 out of 20 mice), while in GSDMD^−/−^ mice, the survival rates were 73% (11 out of 15 mice), 53% (8 out of 15 mice), and 53% (8 out of 15 mice) on the first, second, and third days, respectively (Figure 1A). On the third day after surgery, we conducted an open field test and a novel object recognition test (Figure 1B). The open field test is commonly used to assess the exploratory behavior, anxiety, and motor activity of mice [48], while the novel object recognition test is a behavioral test used in animal research to assess recognition memory and the ability to differentiate between familiar and novel objects [49]. Septic wild-type mice showed a lower discrimination index in the NORT and more border-zone crossings in the OFT compared to the sham mice, indicating that sepsis induced cognitive dysfunction and anxiety (Figure 1C,G).

GSDMD^−/−^ CLP mice showed an improved cognitive function compared to the WT+CLP group (Figure 1C–H). The OFT results indicated a decreasing trend in the border-zone duration in GSDMD^−/−^ CLP mice relative to the WT+CLP group, but no statistically significant difference was observed. Furthermore, an increase in the border-zone duration was observed in the GSDMD^−/−^ CLP mice compared to the GSDMD^−/−^ sham mice, indicating continued anxiety (Figure 1G). Notably, the time spent exploring the two objects also decreased in septic wild-type mice, and the amount of time spent on the familiar object was similar across all four groups, indicating a decrease in motivation in the wild-type CLP mice (Figure 1E,F).

The absence of GSDMD reverses pathological changes in the brain of septic mice.

**Figure 2:**
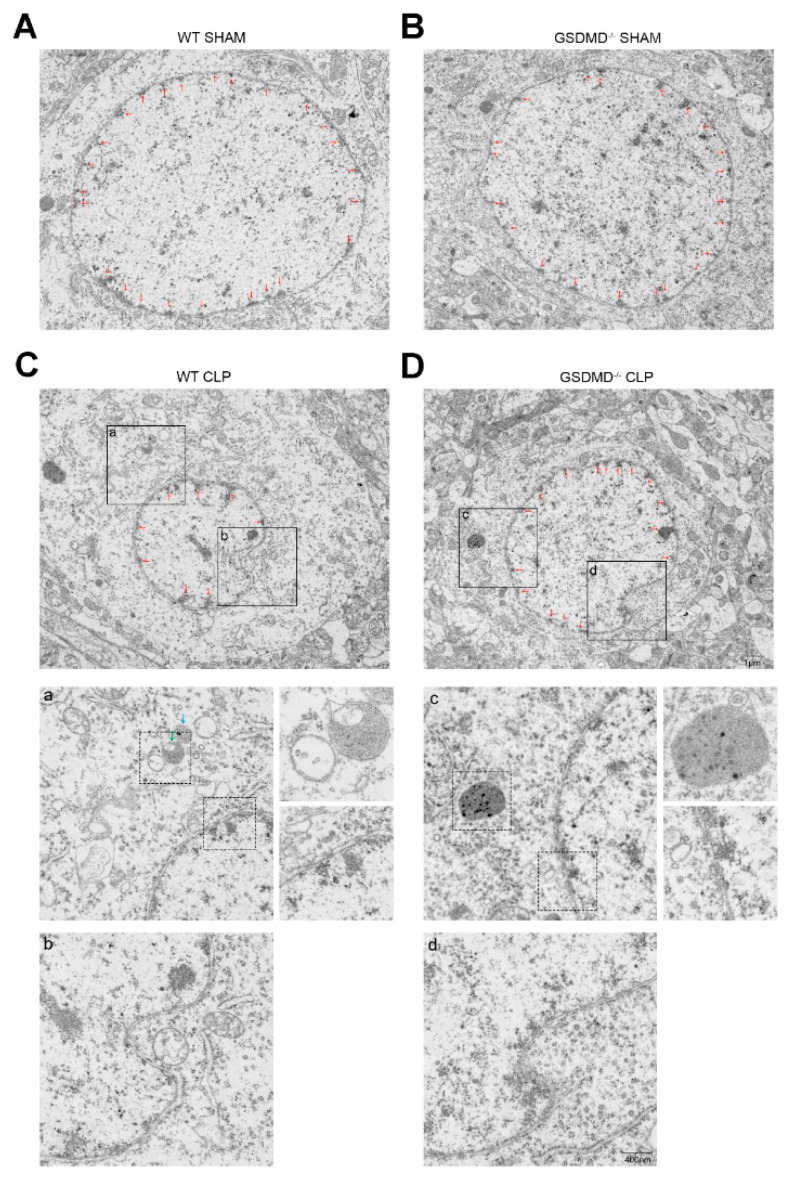
The absence of GSDMD reverses nuclear autophagy in the brain of septic mice. (**A**−**D**) Three days after sepsis onset, representative images of TEM of the nucleus of the wild-type sham group, GSDMD^−/−^ sham group, wild-type CLP group, and GSDMD^−/−^ CLP group, respectively. Scale bar: 1 μm. (**a**−**d**) Representative images of autophagosomes, autolysosomes, and lysosomes. Interruptions of continuity of double neural nuclear membrane (bottom right of diagram (**a**,**c**)) and nuclear membrane collapse in wild-type CLP group and GSDMD^−/−^ CLP group. Scale bar: 400 nm.

Given that the caspase-1 inhibitor has been shown to improve sepsis-related nuclear autophagy [4], we conducted a further analysis of the brain ultrastructure using a TEM. The morphology and structure of neurons were similar and normal in both the wild-type sham group and the GSDMD^−/−^ sham group. The perinuclear heterochromatin had a normal appearance and was abundant in both groups (Figure 2A,B). Nuclear autophagy activation was evident in the neurons of both wild-type CLP and GSDMD^−/−^ CLP mice, as demonstrated by the presence of the indenting double-membrane structure of the nucleus (Figure 2C(b),D(d)). The perinuclear heterochromatin was reduced after CLP in both wild-type and GSDMD^−/−^ mice (Figure 2C,D). Autophagosomes (double-membrane vesicles), autolysosomes (double-membrane structure with some electron-dense contents inside), and lysosomes (single, dense layer of membranes with an electron-dense content inside) increased and gathered in the plasma and near the nuclear (Figure 2C(a),D(c)). However, the number of neurons with an abnormal nuclear morphology was less in the GSDMD^−/−^ CLP mice than that in the wild-type CLP mice.

**Figure 3:**
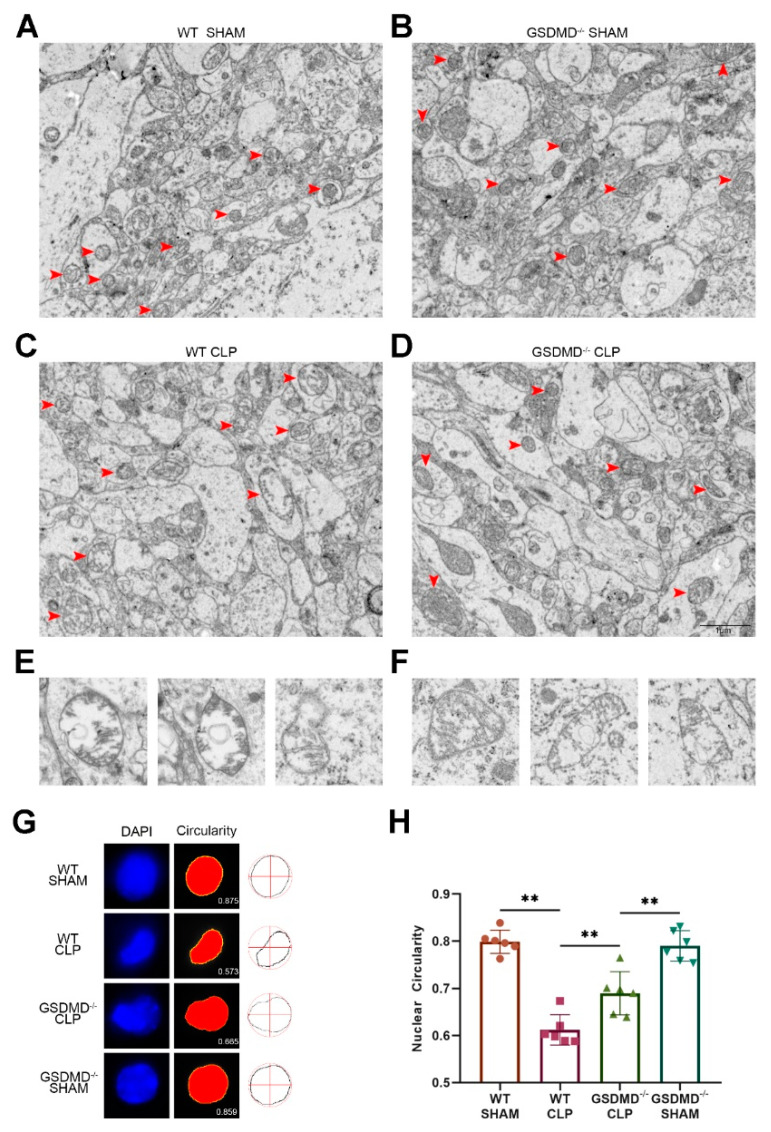
The absence of GSDMD alleviates abnormal morphology of mitochondria and nucleus in the brain of septic mice. (**A**−**D**) TEM images of mitochondrial ultrastructure in the prefrontal cortex on the 3rd day after surgery. Red arrows: mitochondria. Scale bar: 1 μm. (**E**,**F**) Representative images of mitochondria containing double-membranes or edematous mitochondria in wild-type CLP group and GSDMD^−/−^ CLP group. (**G**,**H**) Nuclear circularity analysis of wild-type sham group, GSDMD^−/−^ sham group, wild-type CLP group, and GSDMD^−/−^ CLP group (n = 6 per group). ** *p* < 0.01.

In the sham groups, the mitochondrial morphology was normal, with the crest neatly arranged (Figure 3A,B). In the wild-type CLP mice, disruption of the mitochondrial crest and swelling of some mitochondria were observed (Figure 3C). Interestingly, the presence of bilayer structures in many mitochondria suggested the occurrence of mitophagy (Figure 3E). In comparison to the wild-type CLP mice, the number of swollen mitochondria in the GSDMD^−/−^ CLP mice was smaller, and the crest of the mitochondria was relatively organized (Figure 3D). However, compared to the GSDMD^−/−^ sham mice, the mitochondria in GSDMD^−/−^ CLP mice were smaller in size and the number of mitochondria had increased, indicating that the absence of GSDMD could not fully eliminate hypermetabolism and inflammation (Figure 3D). Interestingly, similar to wild-type CLP mice, many of the mitochondria in GSDMD^−/−^ CLP mice contained double-membrane vesicles (Figure 3F), implying that the absence of GSDMD did not eradicate mitophagy.

To further evaluate nuclear autophagy, we analyzed the circularity of the nucleus using DAPI staining, where the circularity (which ranged from one for a perfect circle to lower values for more convoluted shapes) was found to be reduced in mice with sepsis compared to those in the sham group. Additionally, we noticed that the circularity of the nuclei in GSDMD^−/−^ CLP mice was greater compared to the wild-type CLP mice (Figure 3G,H).

The digestion of LaminB caused by nuclear autophagy is independent to GSDMD.

**Figure 4:**
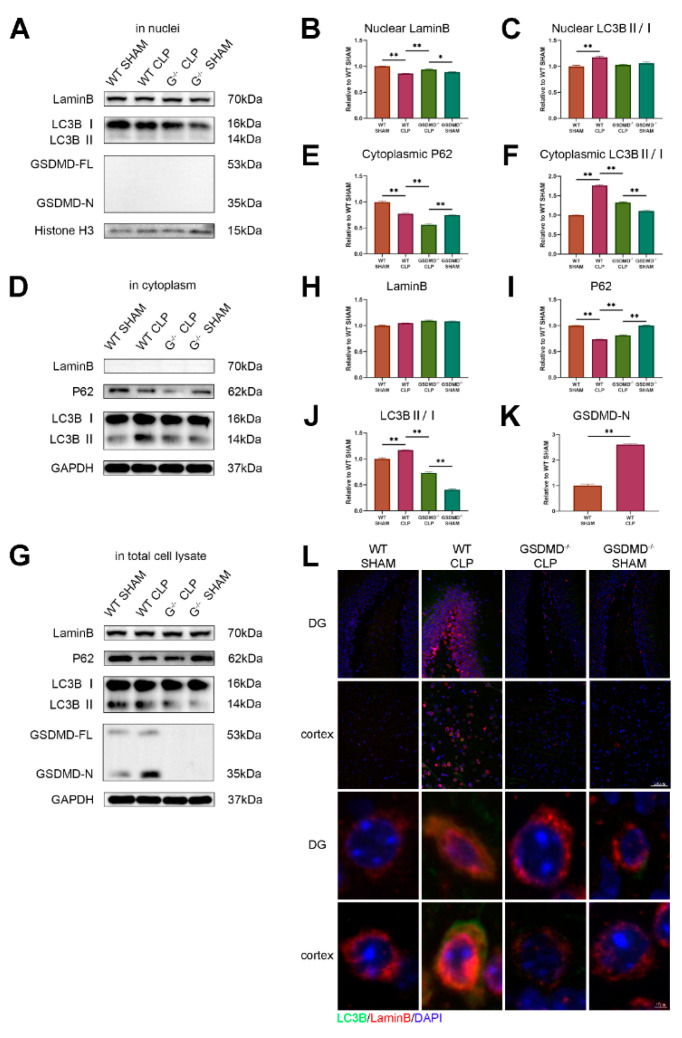
Digestion of LaminB caused by nuclear autophagy is independent of GSDMD. (**A**) Expression of LaminB and LC3BII/I in nuclei is analyzed by Western blots. (**B**,**C**) Statistical analysis of Western blots of A (n = 3 per group). (**D**) Expression of P62 and LC3BII/I in cytoplasm is analyzed by Western blots. (**E**,**F**) Statistical analysis of Western blots of D (n = 3 per group). (**G**) Expression of LaminB, P62, LC3BII/I, and GSDMD-N in whole-cell lysate is analyzed by Western blots. (**H**−**K**) Statistical analysis of Western blots of G (n = 3 per group). (**L**) Representative immunofluorescence staining of LC3B, LaminB, and DAPI in the hippocampus and cortex of wild-type sham group, GSDMD^−/−^ sham group, wild-type CLP group, and GSDMD^−/−^ CLP group. Scale bar: 200 μm and 10 μm. * *p* < 0.05, ** *p* < 0.01.

We then question how sepsis activates nuclear autophagy. In a previous study, LaminB was digested by nuclear autophagy in primary human cells [14]. We then conducted a protein analysis of the hippocampus by separating the nuclear and cytoplasmic proteins, and focused on the examination of LaminB and autophagy markers. We discovered a decrease in the level of LaminB in the nuclei of wild-type CLP mice compared to that in the wild-type sham mice. However, the level of LaminB in the GSDMD^−/−^ CLP mice was not reduced but instead, it was even higher compared to that in GSDMD^−/−^ sham mice (Figure 4A,B). The expression of LaminB in the whole-cell lysate showed no difference between the groups (Figure 4G,H). This may be due to the entry of nuclear LaminB into the cytoplasm not being fully degraded. Further, the content of LaminB in the cytoplasm was too low to be detected (Figure 4D).

In wild-type mice, the LC3BII/I ratio was observed to be higher in the CLP group compared to the sham group in the nuclear component, cytoplasm, and whole-cell lysate (Figure 4A,C,D,F,G,J). This suggested that autophagy was activated in both the nucleus and cytoplasm. In contrast, in the GSDMD^−/−^ mice, the LC3BII/I ratio in the nuclei of septic mice showed no difference when compared to the sham group, while the ratio in the cytoplasm and whole-cell lysate was higher in septic mice than in sham mice, confirming the TEM results that cytoplasmic autophagy was reduced in the GSDMD^−/−^ septic mice compared to wild-type septic mice. In comparison to their respective sham controls, a decrease in the levels of P62 was observed in the cytoplasmic and whole-cell lysates of both the wild-type and GSDMD^−/−^ septic mice. The level of P62 was found to be even lower in wild-type CLP mice compared to GSDMD^−/−^ CLP mice. Additionally, an increase in GSDMD-N was observed in wild-type CLP mice, and it was predominantly located in the cytoplasmic lysate (Figure 4). The results of the fluorescence indicated a significant increase in the expression of LC3B and LaminB in the hippocampus and cortex of wild-type CLP mice. Furthermore, some of the cytoplasmic LC3B was observed to be colocalized with LaminB in the wild-type CLP mice (Figure 4L).

## 4. Discussion

In our study, we discovered that CLP-induced sepsis caused an increase in brain nuclear autophagy. We also found that knocking out GSDMD in mice improved their survival rate and reversed the pathological changes in SAE. These improvements included better cognitive function and a reduction in abnormal nuclear morphology. Interestingly, we did not find any evidence that GSDMD was associated with LaminB digestion induced by nuclear autophagy.

From our TEM analysis of cerebral tissue in septic wild-type mice, we observed a heightened nuclear autophagy in neurons and reduced perinuclear heterochromatin. Additionally, we found that mitophagy was also activated. The autophagosome and lysosome were observed to be concentrated near the nucleus. In comparison, nuclear autophagy was alleviated in neurons and perinuclear heterochromatin was preserved in septic GSDMD^−/−^ mice. Nevertheless, high levels of mitophagy were still observed. The results obtained from TEM were further supported by our analysis of LC3B and P62, which showed the activation of autophagy in both the nuclear and cytoplasmic components during sepsis. However, cytoplasmic autophagy remained high even in the GSDMD^−/−^ CLP mice. During autophagy, the autophagosome fuses with the lysosome, and the contents of the autophagosome are degraded, including p62. Thus, increasing autophagic flux results in decreasing levels of p62 [50].

Additional examination needs to be conducted to ascertain how pyroptosis affects nuclear autophagy in SAE. A recent study demonstrated that GSDMD-N can also be found on the membranes of various intracellular organelles [51]. These discoveries highlight a potential connection between GSDMD, autophagy signaling [10], and mitochondrial damage [52]. Indeed, in the inflammasome-activated neutrophils, GSDMD-N, does not traffic to the plasma membrane, instead, it traffics to autophagosomes and promotes IL-1β release via an autophagy machinery-dependent pathway [10]. In our study, GSDMD-N did not existed in the nuclear component after sepsis, suggesting that GSDMD-N probably did not participate in the formation of the nuclear membrane vesicles or phagosomes in our CLP model. However, sepsis-induced nuclear autophagy was inhibited in GSDMD^−/−^ mice, and the upregulation of LC3B was observed in both cytoplasmic and nuclear components, implying that GSDMD was involved in the process of nuclear autophagy. To further analyze the interaction between LC3B and GSDMD, a more comprehensive study is needed using techniques such as immunoprecipitation and immunofluorescence. LaminB is a substrate of nuclear autophagy [14]. Our study revealed that in sepsis, the degradation of nuclear LaminB could be suppressed in the absence of GSDMD. However, there was no significant difference in LaminB levels within whole-cell lysates across the four groups. These findings suggested that GSDMD was involved in the digestion of nuclear LaminB but not cytoplasmic LaminB during sepsis. One possible explanation was that LaminB was engulfed by autophagic vesicles and translocated to the cytosol where it remained incompletely cleared, or the changes in the LaminB levels in whole-cell lysates might be too subtle to be detected. A reported study [14] indicates that the LC3–LaminB interaction does not downregulate LaminB during starvation, but facilitates its degradation upon oncogenic insults, such as by activated RAS, which confirms this hypothesis.

In addition to LaminB, numerous other nuclear components are expected to bind with LC3 [16], suggesting a vast territory of nuclear autophagy that remains to be uncovered. In a recent study, researchers have uncovered that the protein SIRT1 serves as a newly discovered target for nuclear autophagy during senescence and aging. Direct interaction with LC3 facilitates the protein’s degradation, leading to its relocation from the nucleus to the cytoplasm. Eventually, the autophagosome–lysosome pathway degrades the protein [53]. In sepsis, autophagy has been regarded as a cellular adaptive mechanism for removing damaged proteins and organelles [54]. When sepsis is in its early stage, the initiation of autophagy serves as a defensive mechanism against microbial infection. However, if sepsis becomes severe, an elevated level of autophagy cannot mitigate the excessive inflammatory reaction [55]. On the contrary, autophagy can negatively regulate pyroptosis through the elimination of damage-associated molecular pattern molecules (DAMPs) and pathogen-associated molecular patterns (PAMPs), as well as the clearance of indispensable components in pyroptosis, such as NLRPs [56,57,58]. Nonetheless, it remains unclear in SAE whether nuclear autophagy should be considered beneficial or detrimental. Our study suggests that the enhanced behavioral outcomes observed in GSDMD^−/−^ CLP mice could be attributed to the anti-inflammatory effects resulting from the absence of GSDMD.

## 5. Conclusions

CLP-induced sepsis triggers an increase in brain nuclear autophagy. The absence of GSDMD inhibits nuclear autophagy and improves the behavioral outcomes of SAE. This finding may pave the way for the development of new treatments for SAE that target GSDMD and nuclear autophagy. This study also adds to our understanding of the pathophysiology of SAE, which could lead to improved diagnosis and management of this condition in clinical practice.

## Data Availability

The data sets used and analyzed during the current study are available from the corresponding author upon reasonable request.

## Supplementary Materials

The following supporting information can be downloaded at: https://www.mdpi.com/article/10.3390/brainsci13030478/s1, Figure S1: WB entire membrane; Table S1: List of antibodies used in western blotting and immunofluorescence.
