# Supplementary material for: The Absence of Gasdermin D Reduces Nuclear Autophagy in a Cecal Ligation and Puncture-Induced Sepsis-Associated Encephalopathy Mouse Model
Source: Brain Sci. 2023 Mar 11;13(3):478. doi: 10.3390/brainsci13030478 (PMC10046561; doi:10.3390/brainsci13030478)
Supplement: Supplementary file 1 [file brainsci-13-00478-s001.zip › brainsci-2216591-supplementary.pdf]

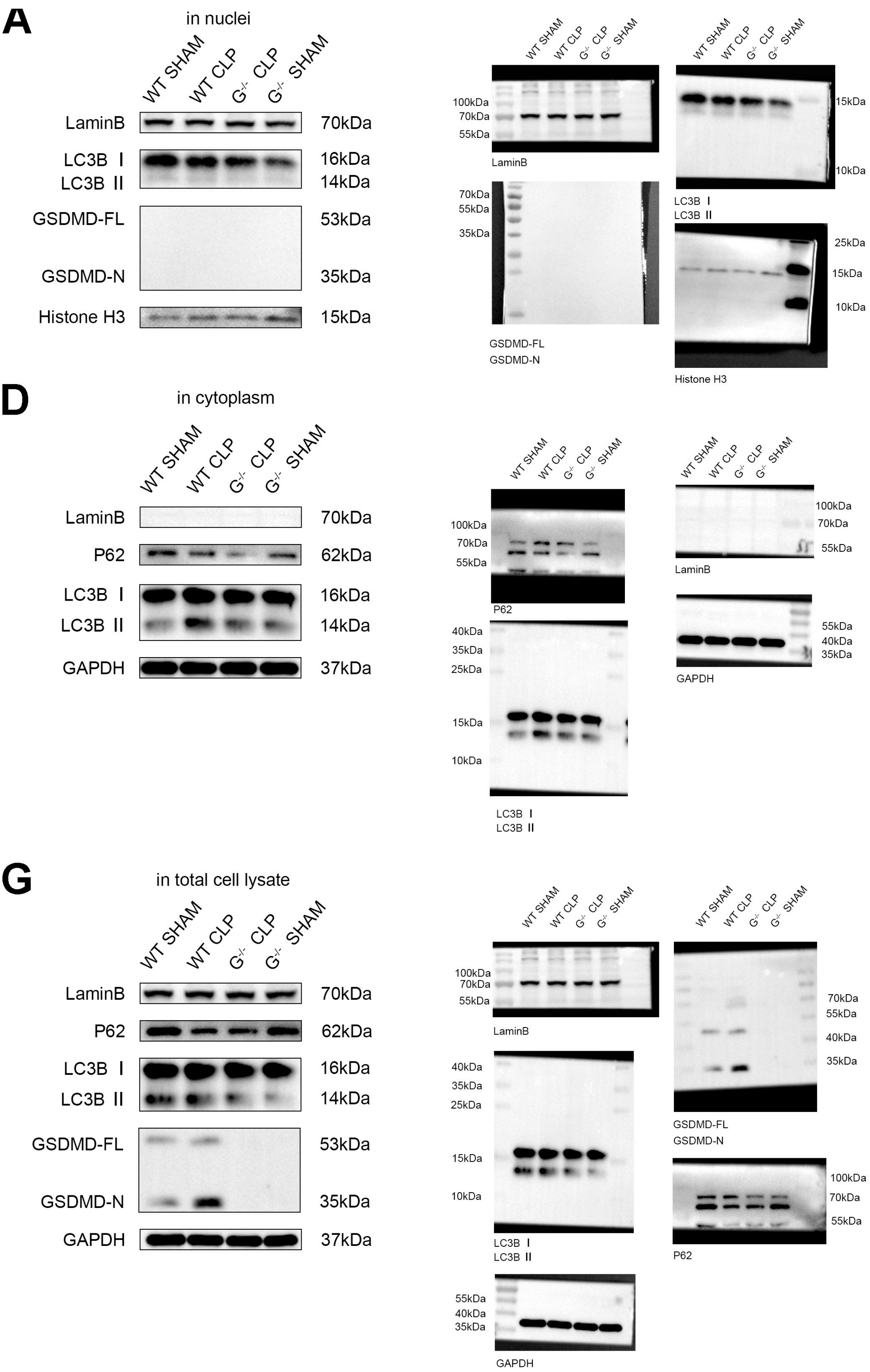

Figure S1. WB entire membrane.

Table S1. List of antibodies used in western blotting and immunofluorescence.

Antibody	Source
GSDMD (sc-393656)	Santa Cruz Biotechnology (United States)
GSDMD-NT (10137S)	Cell Signaling Technology (United States)
LaminB1 (ab8982)	Abcam (United Kingdom)
GAPDH (ab9485)	Abcam (United Kingdom)
LC3B (ab48394)	Abcam (United Kingdom)
Histone H3 (17168-1-AP)	Proteintech (Wuhan, China)
P62 (rabbit, A19700)	Abclonal (Wuhan, China)
488-conjugated donkey polyclonal secondary antibody to Mouse IgG-H&L (715-545-150)	Jackson Immuno Research.
594-conjugated donkey polyclonal secondary antibody to rabbit IgG-H&L (711-585-152)	Jackson Immuno Research.
